# Association between self-perception period of lower urinary tract symptoms and International Prostate Symptom Score: a propensity score matching study

**DOI:** 10.1186/s12894-015-0021-x

**Published:** 2015-04-10

**Authors:** Sung Ryul Shim, Jae Heon Kim, Hoon Choi, Jae Hyun Bae, Hae Joon Kim, Soon-Sun Kwon, Byung Chul Chun, Won Jin Lee

**Affiliations:** Department of Epidemiology and Medical Informatics, Korea University, Seoul, South Korea; Department of Urology, Soonchunhyang University College of Medicine, Seoul Hospital, Seoul, South Korea; Department of Urology, Korea University College of Medicine, Ansan Hospital, Ansan, South Korea; Biomedical Research Center, Seoul National University Bundang Hospital, Seongnam, South Korea; Department of Preventive Medicine, College of Medicine, Korea University, Seoul, South Korea

**Keywords:** Lower urinary tract symptoms, Prostatic hyperplasia, Self-concept

## Abstract

**Background:**

Most studies focusing on progression of BPH have been limited to the relationship between age and BPH progression, and only few studies have focused on the time duration to start treatment. This study aimed to investigate the association between self-perception period (S-PP) of lower urinary tract symptoms (LUTS) and International Prostate Symptom Score (IPSS).

**Methods:**

This study used data from two large-population surveys: a community-based survey and a university hospital outpatient-based interview survey. Both surveys were conducted in male subjects aged 40 years or older who gave consent to the survey questionnaire and voluntarily expressed their intention to participate. Propensity score matching (PSM) was used to organize the population in both surveys into randomized groups to reduce selection bias. After excluding those who had missing values, 483 subjects were assigned to each group by PSM.

**Results:**

The S-PP of LUTS became significantly longer as the severity of LUTS increased. The S-PP was 4.15 years in the mild group, 4.36 years in the moderate group, and 6.23 years in the severe group. These differences were statistically significant. The correlation between S-PP of LUTS and IPSS was measured by partial correlation while controlling for age (correlation coefficient = 0.20, *p* <0.001). Multiple regression analysis after controlling for age revealed that one-year increase in the S-PP of LUTS significantly (*p* <0.001) increased IPSS by 0.322 points.

**Conclusions:**

This study clarified the association between S-PP of LUTS and IPSS in a large-scale population. These findings suggest that, from the perspective of public health, S-PP is an important risk factor for LUTS progression.

## Background

Lower urinary tract symptoms (LUTS) of voiding and storage commonly affect middle-aged men. European EPIC study has estimated that LUTS is present in 62.5% of middle-aged men (voiding symptoms, 25.7%; storage symptoms, 51.3%) [1]. Korean EPIC study has estimated that LUTS is present in 53.7% of middle-aged men (voiding symptoms, 28.5%; storage symptoms, 44.6%) [2].

Benign prostatic hyperplasia (BPH), often causes voiding symptoms, a highly prevalent condition in middle-aged men. In the United States, South Korea, the United Kingdom, and Japan, the prevalence of BPH in age group of 40s to 80s is reported to be 33%, 23%, 41%, and 37%, respectively [3-6]. BPH, representing 80% of causes of geriatric diseases associated with urination, is known to cause decline in sexual function [7] and deterioration in both urination-related [8] and health-related [9] quality of life (QoL). BPH is a progressive disease, with most patients experiencing symptoms worsening over time [10,11]. BPH does not suddenly appear as a disease. It develops slowly in a natural process after subjective perception of LUTS. Therefore, along with age, the self-perception period (S-PP) of LUTS was considered as one of the most important risk factors for BPH [12-14].

Escalating medical expenditures are a major concern in many countries, especially those with populations having high incidence of BPH. In the United States, almost 8 million visits were made with primary or secondary diagnosis of BPH. In 2000, the direct cost of BPH treatment was estimated to be $1.1 billion, exclusive of outpatient pharmaceuticals [15].

From the perspective of public health, identifying risk factors for LUTS is useful to prevent LUTS and to improve QoL. Olmsted County study [16], one of the largest longitudinal studies conducted in America, investigated age as one of many sociodemographic characteristics that may predict the incidence of BPH. Age was also investigated in a study that reanalyzed the same patients of the Olmsted County study [17]. Thus, age is one of the most reliable risk factors for the progression of BPH. Its influence is greater than those of other sociodemographic characteristics. Likewise, most studies focusing on the natural history and progression of BPH have been limited to the relationship between age and BPH progression. In reality, the timing of the first hospital visit after LUTS is different between individuals. In order to explain this more comprehensively, Our previous studies adopted the idea of an S-PP of LUTS from patient’s perspective because BPH is characterized by deep involvement of highly subjective symptoms. These studies showed that the S-PP of LUTS, in addition to age, acted as a major risk factor for LUTS [12-14]. However, results from these studies were insufficient to draw general conclusions due to their small sample sizes, even though individuals were sampled equally from large cities, small- and medium-sized cities, and rural areas. The present study attempted to overcome the limitations of previous studies and determine whether S-PP of LUTS is a risk factor for LUTS using data obtained from a community-based interview survey and an interview survey of university hospital outpatients.

## Methods

### Subjects

The present study used data from two large-population surveys: a community-based interview survey and a university hospital outpatient-based interview survey. Both were conducted with male subjects aged 40 years or older who gave and wrote the informed consents to the survey and voluntarily expressed their intention to participate. This study was approved by the Institutional Review Board of Korea University Ansan Hospital. In order to enhance the validity of International Prostate Symptom Score (IPSS), both surveys excluded the following patients: 1) those who had undergone urological surgery which might affect their IPSS score; 2) those who had received any treatment for BPH or prostate cancer; 3) those who had evidence of neurological condition, un-controlled diabetes mellitus, un-controlled hypertension, history of malignancy, urinary tract infection within 3 months, psychiatric illness with medications and alcohol or substance abuse; 4) those who were taking or had taken any drug for the same complaints.

### Community-based interview survey

One investigator visited senior welfare centers in South Korea between May 2010 and April 2013 and carried out a survey of 1,030 males using the IPSS questionnaire. During this period, the survey was conducted over a total of 36 times in six metropolitan areas of the country: Seoul, Gyeonggido, Incheon, Daejeon, Daegu, and Busan. In total, the study had a sample size of 518 subjects after excluding those described above.

### University hospital outpatients-based survey

Another IPSS questionnaire survey was performed with 2,493 male outpatients who visited university hospitals in South Korea between September 2010 and September 2011. The survey included 20 university hospitals in nine major areas of the country: Seoul, Gyeonggido, Incheon, Daejeon, Daegu, Busan, Gangwondo, Gwangju, and Ulsan. In total, the study had a sample size of 1,278 subjects after excluding those described above.

### Study design and measuring tools

This was a cross-sectional study. To evaluate the factors that might affect the severity of LUTS, the study investigated age, S-PP, and IPSS.

### IPSS questionnaire

The severity of LUTS was measured by IPSS based on the American Urological Association (AUA) symptom index, with one additional question on quality of life. IPSS questionnaire has been translated into many different world languages and adapted based on the circumstances of each country. IPPS questionnaire is now widely used for objective assessment of LUTS [6,18]. The Korean version of the IPSS verified by Choi et al. in terms of relevance and reliability is now the most typical diagnostic instrument for LUTS in Korea [19].

The IPSS questionnaire consisted of eight items, which included seven 6-point scale questions on symptoms (feeling of incomplete emptying, urinary frequency, interrupted stream, urinary urgency, weak urinary stream, urinary hesitancy, and nocturia) and one 7-point scale question on patient’s satisfaction with their urinary condition. Based on the criteria of Barry et al., symptom severity was divided into three groups: mild (a symptom score of 0–7), moderate (8–19), and severe (20–35) [20]. The quality of life or level of satisfaction of LUTS patients was represented by seven grades: “No problem” (0 point = very satisfied), “I’m all right” (1 point), “Somewhat satisfied” (2 points), “Half-satisfied, half-dissatisfied” (3 points), “Somewhat dissatisfied” (4 points), “Distressed” (5 points), and “I can’t stand it” (6 points = very dissatisfied).

### Age questionnaire

Age as an important factor has impact on generation-specific prevalence, IPSS, and S-PP of LUTS. Therefore, this study queried each participant’s date of birth.

### S-PP of LUTS

The S-PP of LUTS was defined as the period between the moment the participant perceived any inconvenience resulting from LUTS (feeling of incomplete emptying, interrupted stream, urgency, weak urinary stream, hesitancy, or nocturia) and the time the interview survey was conducted. The longest periods of any LUTS symptoms were regarded as S-PPs.

### Reliability

Cronbach’s α was 0.652 for the seven 6-point scale questions about symptoms and the one 7-point scale question on satisfaction with urinary conditions, indicating acceptable internal consistency and reliability. Internal consistency with each item excluded did not substantially change the observed value. The reliability of the questionnaire used in this study was estimated to be similar to, or at least not lower than, that in previous studies [14]. This suggested that we used the same method as previous studies. The interview survey of university hospital outpatients was conducted by well-trained professional investigators. The present survey enrolled hospital patients through a formal procedure in compliance with the guidelines of the individual hospitals’ institutional ethics committees.

### Propensity score matching

Propensity score matching (PSM) was used to organize the population in both surveys into randomized groups to reduce selection bias in sampled population. Since the population of this study included two different groups of people, those “who do not visit hospitals” (in the community-based interview survey) and those “who visit hospitals” (in the interview survey of university hospital outpatients), we allowed Berkson’s bias which may result from differences in characteristics between the two groups [21]. PSM typically involves the formation of pairs of treated and untreated subjects with similar propensity score (PS) values. Hence, a logistic regression model was used to calculate and save the predicted probability of the dependent variable and the PS for each observation in the data set. This single score (between 0 and 1) represented the relationship between multiple characteristics and the dependent variable as a single characteristic. Age and S-PP, whose significance was established in previous studies [12-14], were used as independent variables in the analysis.

In this study, in-caliper nearest-neighbor matching proposed by Rosenbaum and Rubin was taken into consideration as a PSM method. Rosenbaum and Rubin suggest use caliper value equal to 0.25 of the standard deviation of the logit of the PS [22]. Accordingly, this study used a caliper of 0.25 times the standard deviation of the PS. Furthermore, one-to-one matching was performed in order to optimize possible effects through several simulations. Excluding those with missing values, 483 subjects were selected from each group using PSM. In this study, the multivariate imbalance measure decreased from 0.50 before PSM to 0.28 after PSM. The implementation of PSM was accordingly evaluated to be appropriate.

### Statistical analysis

In order to examine the association between BPH and related risk factors, we analyzed distribution patterns before and after performing PSM. We performed *t*-test on individual variables to determine whether the confounder was properly controlled between the non-visiting group (for the community-based interview survey) and the visiting group (for the interview survey of university hospital outpatients). For the prevalence of BPH, a frequency analysis was performed on IPSS scores. A one-way ANOVA was carried out to examine the relationships between different BPH severity groups. In an attempt to examine the correlation between the IPSS and the S-PP of LUTS, partial correlation coefficient was measured while controlling for age. A multiple linear regression analysis was conducted to assess how IPSS had changed over a year with respect to risk factors for BPH. In the analysis, independent variables included age and the S-PP of LUTS, both reported to be significant in previous studies [12-14]. The IPSS was used as a dependent variable. The significance of multicollinearity was assessed by comparing the variation inflation factors (VIFs) between independent variables.

All data were presented as mean and standard deviation (SD). Statistical analysis was performed using SPSS version 21.0 software (IBM, New York, NY, USA) with an R module available for PS analysis. All statistics were two-tailed and *p*-values <0.05 were considered to be significant.

## Results

### Characteristics of participants before and after PSM

**Figure 1:**
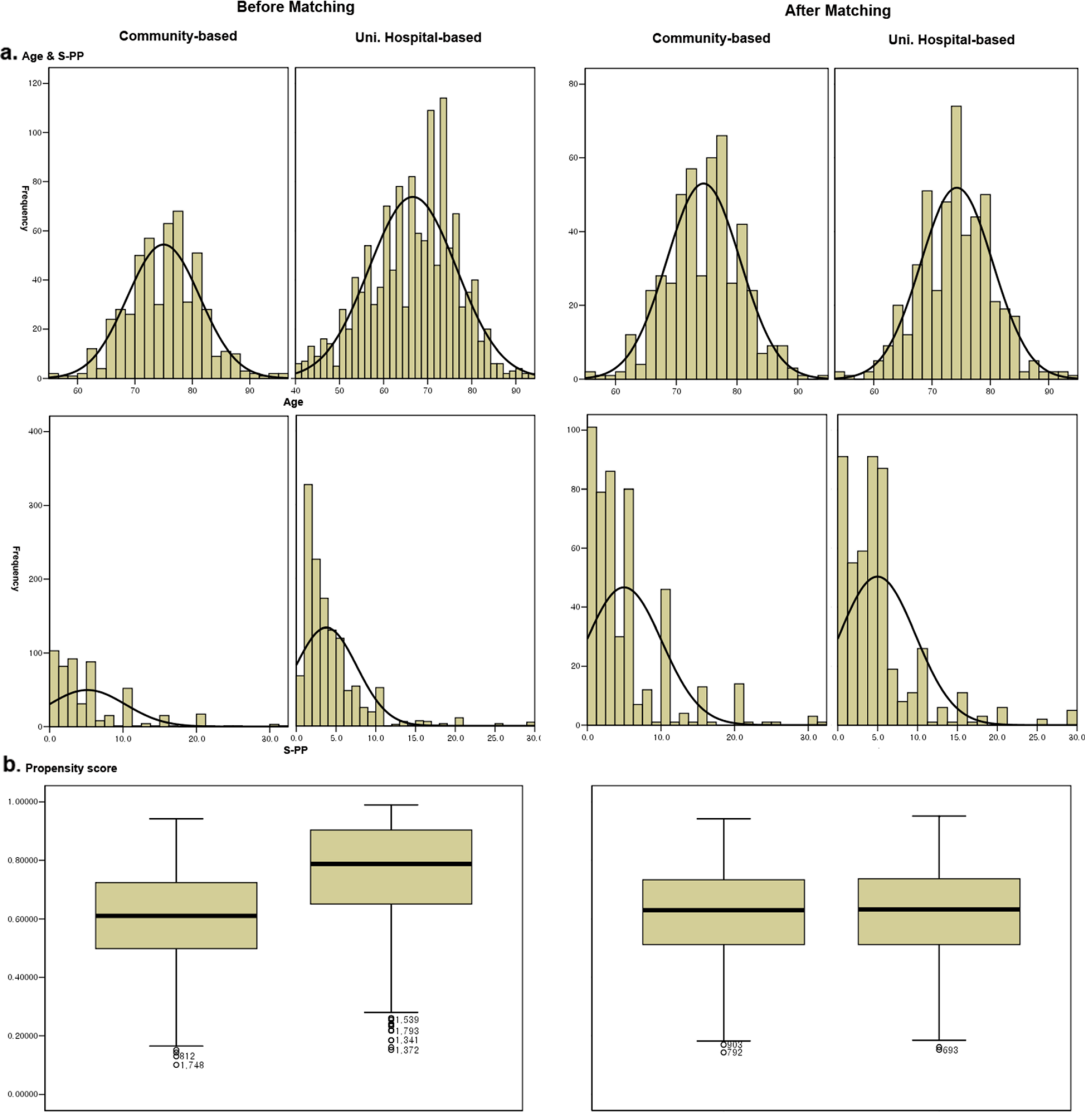
**Comparison of differences of variables between the two groups using histogram (a) and box plot (b) before and after propensity score matching.** Community-based group did not visit hospitals. University hospital outpatients-based group visited hospitals.

With age and S-PP of LUTS as covariates, propensity scores were estimated as the probability of a hospital visit. The distributions of subjects before and after PSM are shown in Figure 1. The distributions of the non-visiting group (subjects in the community-based interview survey) and the visiting group (subjects in the interview survey of university hospital outpatients) before and after PSM were found to be similar to each other in terms of age or S-PP of LUTS (Figure 1a).

Before PSM, the mean propensity scores ranged from 0.46 to 0.72 for the non-visiting group and from 0.64 to 0.92 for the visiting group. The small area of overlap between the two groups indicated that a small number of subjects had similar characteristics. However, when the non-visiting and visiting group (n = 483 per group) were matched one-to-one after PSM, the two groups had the same mean propensity score of approximately 0.6 with a similar distribution (Figure 1b). After PSM, 483 participants were included in each of the non-visiting and visiting groups. The mean age of community-based survey and hospital outpatients’ survey were 74.5 ± 6.06 and 74.3 ± 6.19 years, respectively. There was no statistically significant difference between the two groups in all variables except for IPSS (Table 1).

**Table 1:** **Comparison of variables between the two groups after propensity score matching**

	**Community** ^*^ **n = 483**		**Hospital** ^**†**^ **n = 483**		***P*** ^**‡**^
	**Mean**	**SD**	**Mean**	**SD**	
Age (years)	74.5	6.06	74.3	6.19	0.560
IPSS	15.3	8.16	18.1	7.61	0.000
S-PP (years)	5.0	5.16	5.0	4.79	0.998
PS^§^	0.61	0.15	0.62	0.15	0.618

*Community-based group, those who do not visit hospitals. ^†^University hospital outpatient-based group, those who visit hospitals. ^‡^ *P*-value, student *t*-test analysis. ^§^Propensity score. IPSS, international prostate symptom score; S-PP, self-perception period of lower urinary tract symptoms.

### Prevalence of symptomatic BPH

The distribution of IPSS scores were classified into three groups: mild group (n = 120, 12.4%), moderate group (n = 512, 53.0%), and severe group (n = 334, 34.6%). By age, 87.5% of patients in their 50s, 55.3% of those in their 60s, 53.9% of those in their 70s, and 46.2% of those aged over 80 years belonged to the moderate group. Therefore, as age increased, the proportion of patients in the moderate group was reduced. On the other hand, 12.5% of patients in their 50s, 33.5% of those in their 60s, 34.0% of those in their 70s, and 38.5% of those aged over 80 years belonged to the severe group. Therefore, as age increased, the proportion of patients in the severe group was also increased (Table 2).

**Table 2:** **Age-specific IPSS severity and prevalence of LUTS after propensity score matching**

**IPSS severity**	**Age groups**				
	**50-59**	**60-69**	**70-79**	**over 80**	**Total**
Mild	0 (0)	22 (11.2)	70 (12.1)	28 (15.4)	120 (12.4)
Moderate	7 (87.5)	109 (55.3)	312 (53.9)	84 (46.2)	512 (53.0)
Severe	1 (12.5)	66 (33.5)	197 (34.0)	70 (38.5)	334 (34.6)
Total	8 (100)	197 (100)	579 (100)	182 (100)	966 (100)

Values are numbers with percentages in parentheses. IPSS: international prostate symptom score; LUTS: lower urinary tract symptoms.

### S-PP of LUTS

The S-PP was shown to be 4.15 years for the mild group, 4.36 years for the moderate group, and 6.23 years for the severe group. These differences were statistically significant. A post-hoc Tukey’s test revealed that the severe group showed a significantly longer perception period than the other groups. However, there was no statistical difference between the mild group and the moderate group. Therefore, as the severity of disease increased, the S-PP of LUTS became longer (Table 3).

**Table 3:** **Self-perception period of lower urinary tract symptoms based on severity**

	**n (966)**	**S-PP (years)**		***P*** ^*^
		**Mean**	**SD**	
Mild	120	4.15^†^	3.95	<0.001
Moderate	512	4.36^†^	4.27	
Severe	334	6.23	5.97	

*P-value, one-way analysis of variances. ^†^Same letters indicate no statistical significance based on Tukey’s multiple comparison. S-PP: self-perception period of lower urinary tract symptoms.

### Correlation between S-PP of LUTS and IPSS

The correlation between S-PP and IPSS was measured by Pearson’s partial correlation while controlling for age, which has a significant impact on both variables, with IPSS and S-PP of LUTS as control variables, S-PP of LUTS as an independent variable, and IPSS as a dependent variable. A weak correlation with a correlation coefficient of 0.20 was found (*p* <0.001; Table 4).

**Table 4:** **Partial correlation between self-perception period of LUTS and IPSS**

	**n**	r*	***P***
After propensity score matching	966	0.200	<0.001

*Pearson’s partial correlation coefficient adjusted for age. IPSS: international prostate symptom score; S-PP: self-perception period of lower urinary tract symptoms.

### Association between S-PP of LUTS and IPSS

A multiple linear regression analysis was performed to assess how IPSS had changed over a year with respect to risk factors for LUTS. The IPSS was used as a dependent variable. Result of regression analysis found that a one-year increase in the S-PP of LUTS significantly (*p* <0.001) increased IPSS by 0.322 points and a standardized coefficient (β) of 0.200 (*p* <0.001). Age did not show a statistically significant increase in IPSS. The VIF among independent variables was 1.006. Multicollinearity was not significant (Table 5).

**Table 5:** **Multiple linear regressions analysis of self-perception period of LUTS and IPSS**

	**B** ^*^	**S. E.**	**β** ^**†**^	***P***	
Age (years)	0.013	0.041	0.010	0.75	(n = 966)
S-PP (years)	0.322	0.051	0.200	<0.001	

*Unstandardized coefficient. ^†^Standardized coefficient. IPSS: international prostate symptom score; S-PP: self-perception period of lower urinary tract symptoms.

## Discussion

LUTS is common in elderly men. This was confirmed in the present study. Especially, BPH symptoms become more severe as age increases. This result is similar to those of previous studies [3-6]. BPH can be associated with a number of health-related problems relevant to older men, including increased risk of acute urinary retention, sexual dysfunction, and BPH-related surgery [7,8]. In light of the high prevalence of BPH in older men, increasing life expectancy and retaining a good QoL for older patients requires addressing therapeutic issues and identifying the risk factors of BPH in the general population.

The risk factors for BPH can be seen from two perspectives. The first involves the sociodemographic characteristics of patients, which include age, family history, race, and ambient temperature [10,16,17]. The second includes biological factors, which include age, peak urine flow, prostate volume, PSA, and sense of residual urine [23]. This study placed its focus on sociodemographic characteristics. The self-perception period of LUTS could be considered as one of the most important risk factors for LUTS along with age. Emberton et al. reported that the natural history of LUTS, such as urinary frequency, urgency, nocturia, interrupted stream, weak urinary stream, and sense of residual urine might vary with age [24].

In the correlation analyses of previous studies, the correlation coefficient between age and IPSS was shown to be 0.17 [25] or 0.377 [26]. Age is one of the reliable risk factors for progression of BPH. Influence of age was the greatest among sociodemographic characteristics. Meanwhile, when controlling for age, partial correlation coefficient in the present study between the S-PP of LUTS and IPSS showed a significant correlation (partial correlation coefficient = 0.20, *p* <0.001). These findings suggest that S-PP, in addition to age, is an important risk factor for LUTS progression.

Assessing the progression of BPH by natural history and the Olmsted County study, one of the largest longitudinal studies conducted in America, was focused on age. This study was conducted on 2,115 patients over 42 months. In this study, IPSS was increased by 0.18 points on a yearly basis [16]. In a study that reanalyzed the same patients in the 66th and 92nd month, a 0.3 point [17] increase was observed, respectively. Thus, age is one of the most reliable risk factors for the progression of LUTS. Its influence is the greatest of all sociodemographic characteristics. However, in our study, a regression analysis with age and S-PP revealed that yearly increases of age. IPSS was increased by 0.013 points (*p* = 0.75) as age was increased yearly without statistical significance. Age was not a significant predictor of increase in IPSS severity because both large-population surveys did not control for age-generation distributions. Consequently, almost half of the participants were over 70 years old. We also found that as S-PP of LUTS was increased by 1 year, IPSS would increase by 0.322 points (*P* <0.001), which was a greater change than what was seen for age. The induced regression equation was as follows: IPSS = 14.109 + 0.322 (S-PP) + 0.0 13 (age). In the event when S-PP and age were increased by 1 year, IPSS could be increased by as much as 0.335 points. This result is smaller than the 0.868 points (*p* <0.001) found in a previous study using the same methodology [14]. The results of this regression analysis confirmed the results of correlation analysis described above. That is, from the perspective of public health, the S-PP of LUTS might be a more confident sociodemographic characteristic than age.

Emberton et al. raised the issue that, although the notion of disease progression in BPH is generally accepted, there remains uncertainty about the rate and the determinants of progression. In a review on placebo arms of clinical trials of BPH, progression was observed in terms of increasing prostate volume, decrease in urinary flow rate, and an increase in the future risk of acute urinary retention and surgery. By contrast, symptom score was shown to be improved in placebo groups, probably as a result of the “placebo effect” and the “white coat effect” [27,28]. In reality, however, the time of the first visit to a hospital after perceiving LUTS differs between individuals. The S-PP of symptoms could mean the “self-delayed time” before visiting a health provider. It could be affected by various sociodemographic characteristics. Several studies have shown that several sociodemographic risk factors could affect the “self-delayed time” before the first visit to healthcare provider [13,24,29,30]. The S-PP of LUTS before treatment could have a strong relationship with symptom severity at the time of first medical visit. The present study demonstrated that LUTS development depended on the period of patient’s subjective perception.

The present study has several limitations. Sample selection bias is unavoidable in observational studies. The general characteristics of patients were uncontrollable in both surveys. There were many missing values in both surveys due to sample selection bias. These results were analyzed using two independent variables (age and the S-PP of LUTS) whose significance was established in previous studies [14]. Therefore, it did not represent the whole population of men aged 40 years or older. Furthermore, PSM only accounted for observed covariates. Unobservable covariates cannot be accounted for in the matching procedure. In addition, there was an obvious correlation between IPSS and the S-PP of LUTS. However, a causal relationship could not be observed. Therefore, in the future, a large-scaled active controlled study is needed using a sociodemographically representative population.

## Conclusions

This study clarifies the association between the S-PP of LUTS and IPSS in a large-scale population. These findings suggest that, from the perspective view of public health, S-PP is an important risk factor for LUTS progression. Further longitudinal studies are needed to investigate the real predictive effect of S-PP.

## References

[1] Irwin DE, Milsom I, Hunskaar S, Reilly K, Kopp Z, Herschorn S (2006). Population-based survey of urinary incontinence, overactive bladder, and other lower urinary tract symptoms in five countries: results of the EPIC study. Eur Urol.

[2] Lee YS, Lee KS, Jung JH, Han DH, Oh SJ, Seo JT (2011). Prevalence of overactive bladder, urinary incontinence, and lower urinary tract symptoms: results of Korean EPIC study. World J Urol.

[3] Girman CJ, Epstein RS, Jacobsen SJ, Guess HA, Panser LA, Oesterling JE (1994). Natural history of prostatism: impact of urinary symptoms on quality of life in 2115 randomly selected community men. Urology.

[4] Lee E, Yoo KY, Kim Y, Shin Y, Lee C (1998). Prevalence of lower urinary tract symptoms in Korean men in a community-based study. Eur Urol.

[5] Trueman P, Hood SC, Nayak US, Mrazek MF (1999). Prevalence of lower urinary tract symptoms and self-reported diagnosed ‘benign prostatic hyperplasia’, and their effect on quality of life in a community-based survey of men in the UK. BJU Int.

[6] Tsukamoto T, Kumamoto Y, Masumori N, Miyake H, Rhodes T, Girman CJ (1995). Prevalence of prostatism in Japanese men in a community-based study with comparison to a similar American study. J Urol.

[7] Gacci M, Bartoletti R, Figlioli S, Sarti E, Eisner B, Boddi V (2003). Urinary symptoms, quality of life and sexual function in patients with benign prostatic hypertrophy before and after prostatectomy: a prospective study. BJU Int.

[8] Yoshimura K, Arai Y, Ichioka K, Terada N, Matsuta Y, Okubo K (2002). Symptom-specific quality of life in patients with benign prostatic hyperplasia. Int J Urol: Off J Japanese Urol Assoc.

[9] Hunter DJ, McKee M, Black NA, Sanderson CF (1995). Health status and quality of life of British men with lower urinary tract symptoms: results from the SF-36. Urology.

[10] Jacobsen SJ, Jacobson DJ, Girman CJ, Roberts RO, Rhodes T, Guess HA (1999). Treatment for benign prostatic hyperplasia among community dwelling men: the Olmsted county study of urinary symptoms and health status. J Urol.

[11] McConnell JD, Roehrborn CG, Bautista OM, Andriole GL, Dixon CM, Kusek JW (2003). The long-term effect of doxazosin, finasteride, and combination therapy on the clinical progression of benign prostatic hyperplasia. N Engl J Med.

[12] Kim JH, Ham BK, Shim SR, Lee WJ, Kim HJ, Kwon SS (2013). The association between the self-perception period of overactive bladder symptoms and overactive bladder symptom scores in a non-treated population and related sociodemographic and lifestyle factors. Int J Clin Pract.

[13] Kim JH, Shim SR, Lee WJ, Kim HJ, Kwon SS, Bae JH (2012). Sociodemographic and lifestyle factors affecting the self-perception period of lower urinary tract symptoms of international prostate symptom score items. Int J Clin Pract.

[14] Shim SR, Kim JH, Kim KH, Yoon SJ, Lee WJ, Kim HJ (2012). Association between the self-perception period of lower urinary tract symptoms and the international prostate symptom score. Urol Int.

[15] Wei JT, Calhoun E, Jacobsen SJ (2008). Urologic diseases in america project: benign prostatic hyperplasia. J Urol.

[16] Jacobsen SJ, Girman CJ, Guess HA, Rhodes T, Oesterling JE, Lieber MM (1996). Natural history of prostatism: longitudinal changes in voiding symptoms in community dwelling men. J Urol.

[17] Sarma AV, Jacobsen SJ, Girman CJ, Jacobson DJ, Roberts RO, Rhodes T (2002). Concomitant longitudinal changes in frequency of and bother from lower urinary tract symptoms in community dwelling men. J Urol.

[18] Sagnier PP, MacFarlane G, Richard F, Botto H, Teillac P, Boyle P (1994). Results of an epidemiological survey using a modified american urological association symptom index for benign prostatic hyperplasia in France. J Urol.

[19] Choi HR CW, Shim BS, Kwon SW, Hong SJ, Chung BH, Sung DH (1996). Translation validity and reliability of IPSS Korean version. Korean J Urol.

[20] Barry MJ, Fowler FJ, O’Leary MP, Bruskewitz RC, Holtgrewe HL, Mebust WK (1992). The american urological association symptom index for benign prostatic hyperplasia. The measurement committee of the american urological association. J Urol.

[21] Berkson J (1946). Limitations of the application of fourfold table analysis to hospital data. Biometrics.

[22] D’Agostino RB (1998). Propensity score methods for bias reduction in the comparison of a treatment to a non-randomized control group. Stat Med.

[23] Stuart EA (2010). Matching methods for causal inference: A review and a look forward. Stat Sci.

[24] Emberton M, Cornel EB, Bassi PF, Fourcade RO, Gomez JM, Castro R (2008). Benign prostatic hyperplasia as a progressive disease: a guide to the risk factors and options for medical management. Int J Clin Pract.

[25] Lee ELC, Kim YI, Shin YS (1995). Estimation of benign prostatic hyperplasia prevalence in Korea: an epidemiological survey using international prostatic symptom score (IPSS) in Yonchon county. Korean J Urol.

[26] Cho KSJM, Lim DJ, Son HC, Park SK, Yoo KY, Kim HH (2001). Epidemiologic survey using international prostate symptom score of lower urinary tract symptoms in elderly men above 40 years old in Seoul area. Korean J Urol.

[27] Alwan H, Pruijm M, Ponte B, Ackermann D, Guessous I, Ehret G (2014). Epidemiology of masked and white-coat hypertension: the family-based SKIPOGH study. PLoS One.

[28] Emberton M, Fitzpatrick JM, Garcia-Losa M, Qizilbash N, Djavan B (2008). Progression of benign prostatic hyperplasia: systematic review of the placebo arms of clinical trials. BJU Int.

[29] Coyne KS, Kaplan SA, Chapple CR, Sexton CC, Kopp ZS, Bush EN (2009). Risk factors and comorbid conditions associated with lower urinary tract symptoms: EpiLUTS. BJU Int..

[30] Crawford ED, Wilson SS, McConnell JD, Slawin KM, Lieber MC, Smith JA (2006). Baseline factors as predictors of clinical progression of benign prostatic hyperplasia in men treated with placebo. J Urol.

